# Polycrystalline κ-Ga_2_O_3_ on Si(100) substrates with GZO buffer layers

**DOI:** 10.1039/d6ra00464d

**Published:** 2026-03-25

**Authors:** Yoshiaki Hirai, Htet Su Wai, Toshiyuki Kawaharamura, Noriaki Ikenaga, Osamu Ueda, Hiroyuki Nishinaka

**Affiliations:** a Department of Electronics, Kyoto Institute of Technology Kyoto 606-8585 Japan m4621034@edu.kit.ac.jp; b School of Systems Engineering, Kochi University of Technology Kochi 782-8502 Japan; c Department of Electrical and Electronic Engineering, Kanazawa Institute of Technology 7-1 Ohgigaoka Nonoichi Ishikawa 921-8501 Japan; d Meiji Renewable Energy Laboratory, Meiji University 1-1-1 Higashimita, Tama-ku Kawasaki Kanagawa 214-8571 Japan; e Faculty of Electrical Engineering and Electronics, Kyoto Institute of Technology Kyoto 606-8585 Japan nishinaka@kit.ac.jp

## Abstract

Kappa-phase gallium oxide (κ-Ga_2_O_3_) is an emerging piezoelectric semiconductor with potential applications in radio-frequency devices. However, heteroepitaxial growth of κ-Ga_2_O_3_ on silicon substrates remains challenging owing to large lattice mismatch and interfacial oxidation. This study demonstrates the growth of polycrystalline κ-Ga_2_O_3_ thin films deposited by mist chemical vapor deposition (mist CVD) on Si(100) substrates using Ga-doped ZnO (GZO) buffer layers. Structural characterization *via* X-ray diffraction, scanning electron microscopy, and transmission electron microscopy reveals that κ-Ga_2_O_3_ films exhibit a *c*-axis oriented polycrystalline structure with random in-plane orientations, which yields isotropic properties that are advantageous for device applications. A ZnGa_2_O_4_ intermediate layer is identified at the κ-Ga_2_O_3_/GZO interface, which plays a critical role in phase stabilization. These findings indicate that polycrystalline κ-Ga_2_O_3_ on Si substrates represents a promising platform for piezoelectric semiconductor devices.

## Abstract 2

Polycrystalline κ-Ga_2_O_3_ was successfully deposited on a Si substrate using a GZO buffer layer. Furthermore, it was revealed that a ZnGa_2_O_4_ transition layer forms during the growth process.

## Graphical abstract

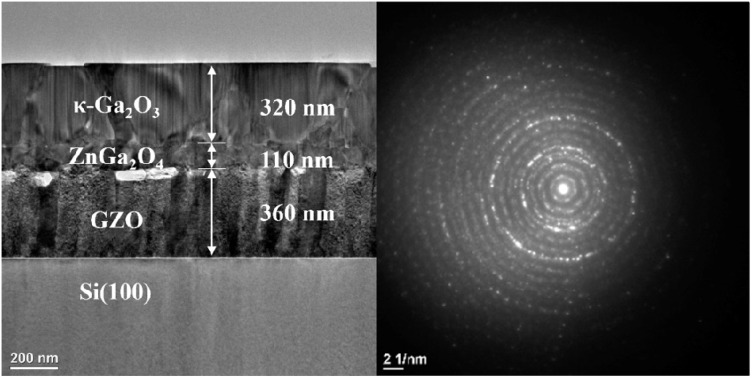

## Introduction

Ultra-wide bandgap (UWBG) semiconductors with bandgaps exceeding 4 eV have gained traction for use in high-power, high-frequency, and high-temperature electronic devices. Among these materials, gallium oxide (Ga_2_O_3_) has emerged as a promising candidate owing to its excellent material properties, including a wide bandgap of approximately 4.9 eV and high breakdown electric field exceeding 8 MV cm^−1^.^1^ Ga_2_O_3_ exists in five distinct polymorphs: α, β, γ, δ, and κ (also referred to as ε). The β-phase is the most thermodynamically stable and has been extensively examined for power device applications.^2,3^ By contrast, the metastable phases, particularly κ(ε)-Ga_2_O_3_, have received relatively limited attention despite their distinctive crystal structures and functional properties.

The κ(ε)-Ga_2_O_3_ polymorph crystallizes in an orthorhombic structure (space group *Pna*2_1_) and is predicted to exhibit strong spontaneous polarization along the *c*-axis,^4^ making it a promising material for piezoelectric applications such as high-electron-mobility transistors (HEMTs) and surface acoustic wave (SAW) devices.^5,6^ Chen *et al.* recently demonstrated strong piezoelectricity in ε-Ga_2_O_3_ thin films with *d*_33_ ≈ 10.8–11.2 pm V^−1^—approximately twice that of AlN—and successfully fabricated SAW resonators operating in the GHz range, making κ-Ga_2_O_3_ a promising candidate for radio-frequency applications.^7^ The pseudo-hexagonal arrangement of oxygen atoms on the κ-Ga_2_O_3_(001) surface enables heteroepitaxial growth on substrates with hexagonal or pseudo-hexagonal symmetry. High-quality epitaxial κ-Ga_2_O_3_ films have been fabricated on diverse single-crystal substrates^4,8–12^ employing diverse deposition techniques including halide vapor phase epitaxy (HVPE),^8^ metal–organic chemical vapor deposition (MOCVD),^9^ mist chemical vapor deposition (mist CVD),^10–12^ molecular beam epitaxy (MBE),^13^ and pulsed laser deposition (PLD).^14^ These epitaxial films typically exhibit characteristic 120° rotational domain structures resulting from the pseudo-hexagonal symmetry of κ-Ga_2_O_3_(001) surface, as observed by Cora *et al. via* plan-view TEM analysis.^15^

Although these single-crystal substrates have supported foundational studies and proof-of-concept devices, integration with silicon substrates is essential for practical applications owing to cost, scalability, and CMOS compatibility. Prior studies demonstrated κ-Ga_2_O_3_ growth on Si using epitaxial buffer layers, such as AlN or Mo,^16,17^ which can form single-crystal templates on Si despite the large lattice mismatch.

For practical piezoelectric device applications, crystallographic isotropy is crucial for design flexibility, as anisotropic properties can limit device orientation and layout options. These ordered 120° rotational domains can provide partial isotropy by averaging anisotropic elastic properties over three discrete orientations.^18^ Building on this observation, an alternative polycrystalline approach using fully randomized films with [001] texture but random in-plane orientations can yield more complete isotropy by averaging over all in-plane orientations. One approach to forming polycrystalline Ga_2_O_3_ is to deposit a thin film at low temperature followed by post-deposition thermal annealing.^19^ However, because κ-Ga_2_O_3_ is a metastable phase, achieving single-phase growth remains challenging. To address this issue, polycrystalline κ-Ga_2_O_3_ films can instead be fabricated using polycrystalline buffer layers that naturally develop *c*-axis texture on amorphous SiO_2_/Si interfaces, thereby promoting phase stabilization during growth. However, key questions remain regarding phase stability, structural uniformity, and interfacial reactions in polycrystalline κ-Ga_2_O_3_ on Si substrates. To address these open questions, this study presents, for the first time, polycrystalline κ-Ga_2_O_3_ growth on Si(100) using a GZO buffer layer—an approach that offers a simpler alternative to epitaxial methods employing AlN or Mo buffer layers and inherently yields isotropic film properties advantageous for piezoelectric device applications.

Irrespective of the growth approach, interface control is critical for achieving phase-pure κ-Ga_2_O_3_. During high-temperature deposition, unintended interfacial reactions can generate β-Ga_2_O_3_ or other secondary phases, which can degrade piezoelectric properties.^20^ Therefore, the choice of buffer layer material and its chemical compatibility with both the substrate and κ-Ga_2_O_3_ overlayer are crucial. Zinc oxide (ZnO) and related materials, with their hexagonal wurtzite structure, represent promising candidates for templating *c*-axis-oriented κ-Ga_2_O_3_ growth.

In this study, we investigate the growth of κ-Ga_2_O_3_ thin films deposited by mist chemical vapor deposition (mist CVD) on Si(100) substrates using Ga-doped ZnO (GZO) buffer layers. The mist CVD technique offers several advantages, including low-cost equipment, atmospheric pressure operation, and high sensitivity to substrate surface conditions, which enables selective nucleation and growth. Furthermore, mist CVD demonstrates a strong compatibility with the formation and stabilization of metastable Ga_2_O_3_ phases.^21,22^ The choice of GZO as a buffer layer is motivated by its hexagonal wurtzite structure and the potential for *c*-axis-oriented growth, which can provide a suitable template for κ-Ga_2_O_3_ deposition. In addition, due to the presence of the amorphous SiO_2_ layer at the Si interface, GZO is expected to grow as an in-plane randomly oriented polycrystalline film. Consequently, κ-Ga_2_O_3_ formed on the GZO/Si template may inherit this in-plane structural disorder, resulting in polycrystalline growth. This is expected to result in effectively isotropic behaviour in the SAW device, as polycrystalline thin films generally exhibit a higher degree of in-plane isotropy than conventionally grown epitaxial films due to the random orientation of grains.

We systematically characterize the structural properties of the resulting κ-Ga_2_O_3_ films *via* X-ray diffraction (XRD), and transmission electron microscopy (TEM) is employed to elucidate the crystal structure, phase composition, and interfacial characteristics. Additionally, scanning transmission electron microscopy (STEM) and energy-dispersive X-ray spectroscopy (EDX) were performed to analyse the elemental composition of the sample. The insights gained from this study contribute to a better understanding of κ-Ga_2_O_3_ growth mechanisms on Si substrates and the potential of polycrystalline κ-Ga_2_O_3_ for piezoelectric device applications.

## Experimental section

First, a Ga-doped ZnO (GZO) buffer layer was deposited onto a Si (100) substrate with a native oxide layer using RF magnetron sputtering. A 4-inch GZO target with a composition of ZnO : Ga_2_O_3_ = 94.3 : 5.7 wt% was applied for the deposition. Prior to sputtering, the substrate was preheated at 150 °C for 1 hour to remove residual moisture and enhance film adhesion. During the deposition, high-purity argon gas was introduced into the chamber at a flow rate of 30 sccm as the working gas, regulated by a mass flow controller. The sputtering pressure, RF power, and substrate temperature were maintained at 1 Pa, 60 W, and 150 °C, respectively. The resulting GZO film thickness was approximately 350 nm after 102 min of sputtering.

Next, κ-Ga_2_O_3_ thin films were grown on GZO/Si (100) substrates by mist CVD. The precursor solution comprised of gallium acetylacetonate (Ga(C_5_H_7_O)_3_) dissolved in deionized water with 1% hydrochloric acid (HCl), where HCl was introduced to ensure complete dissolution of the compound. The precursor solution was atomized using ultrasonic transducers operating at 2.4 MHz and transported to the substrate using N_2_ carrier gas. The film deposition temperature was kept at 760 °C, and deposition time was 30 min. The Ga concentration in the precursor solution was 0.025 mol L^−1^, and the N_2_ flow rate was set to 7.4 L min^−1^. These conditions enable precise control of the growth rate and contribute to the improvement of crystalline quality.^23^

X-ray diffraction (XRD, Bruker D8 Discover) measurements were performed using a CuKα (*λ* = 1.5405 Å) operating at 40 kV and 40 mA as the X-ray source to investigate the crystal structure and orientation relationship of κ-Ga_2_O_3_. Furthermore, cross-sectional and plan-view transmission electron microscopy (TEM, FEI TecnaiF20X) observations, including selected area electron diffraction (SAED) patterns and high-resolution imaging, were performed to examine microstructure and interfacial characteristics, and the measurements were conducted by Ion Technology Center Co., Ltd.

The SAED pattern was obtained using a selected-area aperture with a diameter of 200 nm. Additionally, scanning transmission electron microscopy (STEM, JEOL JEM-2100F) and energy-dispersive X-ray spectroscopy (EDX, JEOL EX-24063JCT) were performed to analyse the elemental composition of the sample.

## Results and discussion

**Fig. 1:**
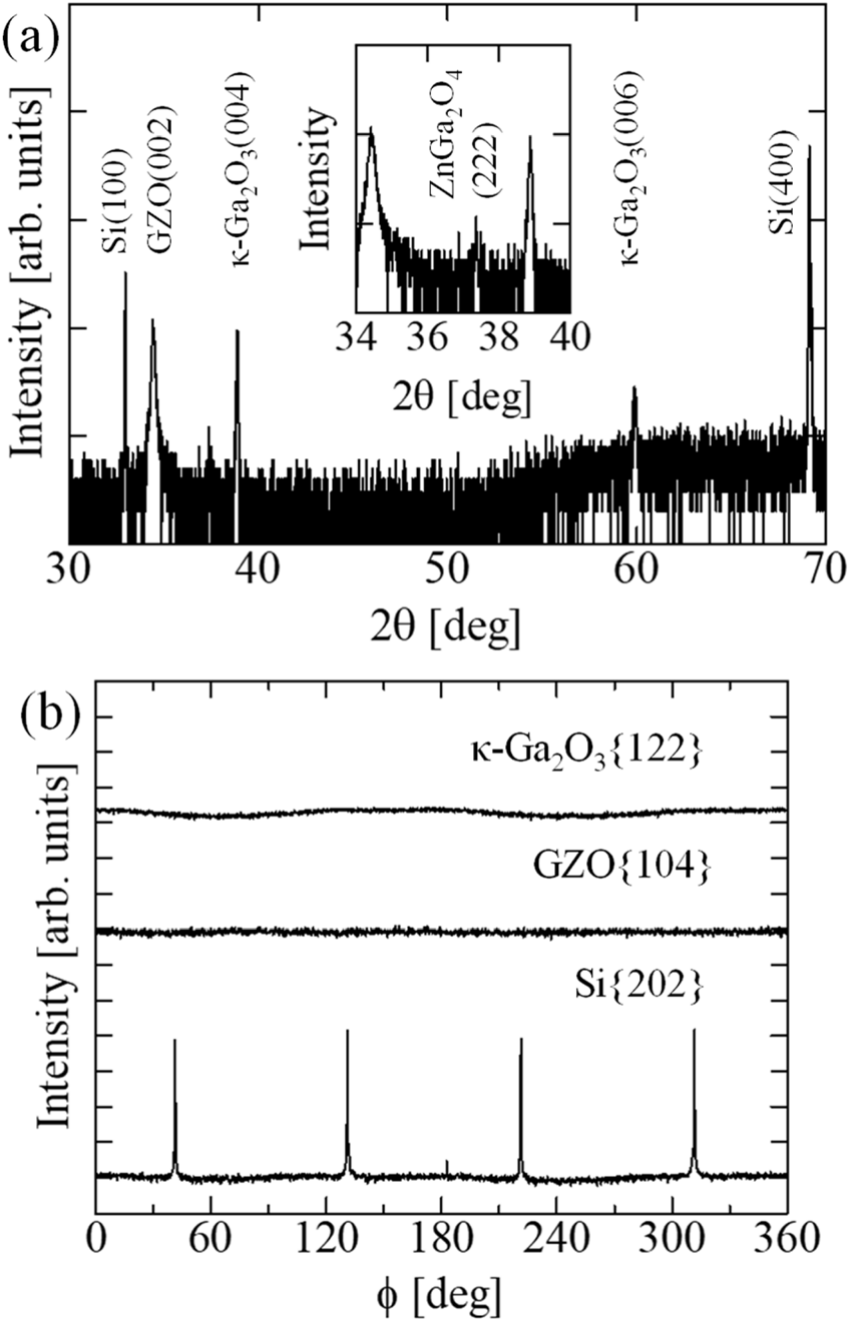
(a) XRD 2*θ*–ω scan of κ-Ga_2_O_3_ grown on GZO/Si. (b) XRD φ-scan of three layers.

We first investigated the crystal structure and orientation relationships of κ-Ga_2_O_3_ thin film grown on GZO/Si(100) substrate *via* X-ray diffraction measurements. Fig. 1(a) shows the XRD 2*θ*–ω scan of the as-grown sample. The diffraction peaks at 2*θ* = 38.9° and 59.9° correspond to (004) and (006) reflections of κ-Ga_2_O_3_, respectively, indicating that [001]-oriented growth on the GZO/Si(100) substrate. The diffraction peak at 2*θ* = 34.4° and 2*θ* = 33.0° correspond to GZO (002) and Si(100) reflections, respectively. The presence of only (00l) reflections for κ-Ga_2_O_3_ and GZO confirms that both layers have preferential *c*-axis orientation perpendicular to the substrate. Notably, a small peak at 2*θ* = 37.4° is observed, corresponding to ZnGa_2_O_4_ (222). This suggests the formation of a ZnGa_2_O_4_ intermediate layer between GZO and κ-Ga_2_O_3_, which will be discussed in detail below based on TEM observations. Furthermore, the absence of diffraction peaks corresponding to other Ga_2_O_3_ polymorphs indicates that κ-Ga_2_O_3_ was grown in a single-phase form.

To investigate the in-plane orientation relationships, φ-scans measurements were performed for κ-Ga_2_O_3_{122}, GZO{104}, and Si{202} reflections, as shown in Fig. 1(b). The Si{202} reflection exhibits four peaks separated by 90°. By contrast, φ-scans of GZO{104} and κ-Ga_2_O_3_{122} show no characteristic peaks. With respect to GZO, this absence of peaks indicates that the buffer layer comprises *c*-axis-oriented polycrystalline grains with random in-plane orientation, which is expected for ZnO-type films grown on amorphous SiO_2_ native oxide on Si substrate. Similarly, the κ-Ga_2_O_3_ {122} φ-scan shows no characteristic peaks, despite the fact that epitaxial κ-Ga_2_O_3_ films typically exhibit either 4 or 12 distinct peaks depending on the presence of rotation domains.^14,24^ This indicates that the κ-Ga_2_O_3_ thin film also comprises randomly oriented grains in the film plane. The polycrystalline nature of GZO and κ-Ga_2_O_3_ layers suggests that the in-plane epitaxial relationship is not established owing to the presence of amorphous SiO_2_ at the Si interface. Although the GZO buffer layer provides a hexagonally arranged oxygen template for *c*-axis-oriented nucleation of κ-Ga_2_O_3_, it does not define a specific in-plane orientation. These results demonstrate that κ-Ga_2_O_3_ grown on polycrystalline GZO(001) buffer layers exhibits a highly textured polycrystalline structure, with a preferential [001] orientation perpendicular to the substrate but random in-plane orientations.

**Fig. 2:**
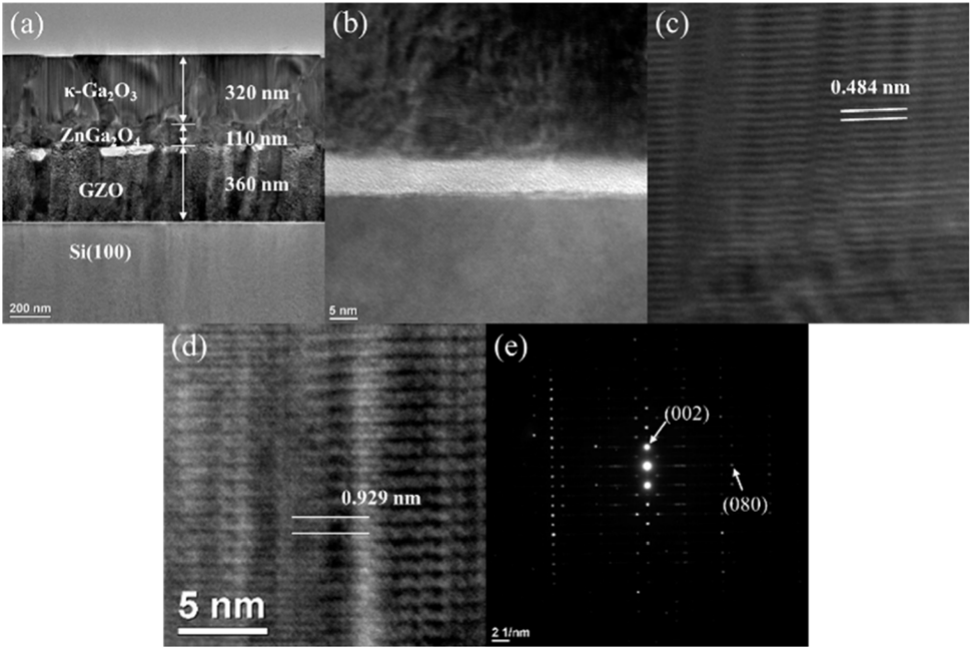
(a) Cross-sectional TEM image of κ-Ga_2_O_3_/GZO/Si. (b) High-resolution TEM images of the GZO/Si interface, (c) ZnGa_2_O_4_ inter layer, and (d) κ-Ga_2_O_3_ film. (e) SAED pattern obtained from κ-Ga_2_O_3_ layer in cross-sectional view.

To further investigate the polycrystalline structure and the layer formation mechanisms, we performed detailed TEM observations. Fig. 2(a) shows a cross-sectional TEM image of κ-Ga_2_O_3_/GZO/Si(100) structure. The thicknesses of κ-Ga_2_O_3_ and GZO were 320 and 360 nm, respectively. A transition layer with distinct bright contrast is observed between the GZO and κ-Ga_2_O_3_ layers, with a thickness of approximately 110 nm. As the κ-Ga_2_O_3_ layer grows away from this transition layer, a characteristic columnar structure develops, which is associated with the growth of polycrystalline grains with [001] orientation.

Fig. 2(b) shows a high-resolution TEM image of the GZO/Si interface. A thin amorphous layer approximately 5–6-nm thick is visible at the interface, corresponding to native SiO_2_ formed on the Si substrate. The GZO layer exhibits polycrystalline microstructure immediately above the amorphous interface, indicating random nucleation without a defined in-plane orientation. Although individual grains maintain *c*-axis orientation perpendicular to the substrate, their in-plane orientations are not correlated owing to the absence of crystallographic templating from the underlying amorphous SiO_2_. This observation confirms that the polycrystalline nature of the GZO buffer layer originates from nucleation on the amorphous SiO_2_, which prevents establishment of an epitaxial relationship with the Si(100) substrate. This is consistent with the absence of characteristics peaks in the GZO{104} φ-scan measurement (Fig. 1(b)).

Fig. 2(c) shows a high-resolution image of the transition layer between GZO and κ-Ga_2_O_3_. The lattice spacing measured in this region is 0.484 nm, which align closely with the (111) plane spacing of ZnGa_2_O_4_ (*d*_111_ = 0.481 nm).^25^ The XRD 2*θ*–ω peak of ZnGa_2_O_4_(222) indicates that ZnGa_2_O_4_ is transiently formed at GZO interface during the growth process of κ-Ga_2_O_3_. This identification is consistent with the small XRD peak observed at 2*θ* = 37.4 corresponding to ZnGa_2_O_4_(222). The formation of intermediate layers at the interface between κ-Ga_2_O_3_ and underlying substrates or buffer layers is a commonly observed phenomenon. Previous studies have reported the formation of various phases, including β-Ga_2_O_3_.^15,20^ The growth process of the ZnGa_2_O_4_ transition layer will be discussed later based on the results of STEM/EDX analysis.

Fig. 2(d) shows a high-resolution image of the κ-Ga_2_O_3_ layer. The lattice spacing measured adjacent lattice fringes is 0.929 nm, which aligns with the (001) place spacing of κ-Ga_2_O_3_ (*d*_001_ = 0.928 nm).^26^

Fig. 2(e) shows the SAED pattern obtained from κ-Ga_2_O_3_ layer in a cross-sectional view. The diffraction spots are aligned along the [001] direction, forming a single-crystal-like pattern. This appearance is attributed to the limited sample volume probed by the selected area aperture in the cross-sectional geometry, which contains only a small number of columnar grains with similar [001] orientations perpendicular to the substrate. The cross-sectional SAED primarily reflects the out-of-plane texture rather than the in-plane orientation distribution. A more definitive analysis of the in-plane structure, requiring sampling a larger area multiple grains, is provided by plan-view TEM observations discussed below.

**Fig. 3:**
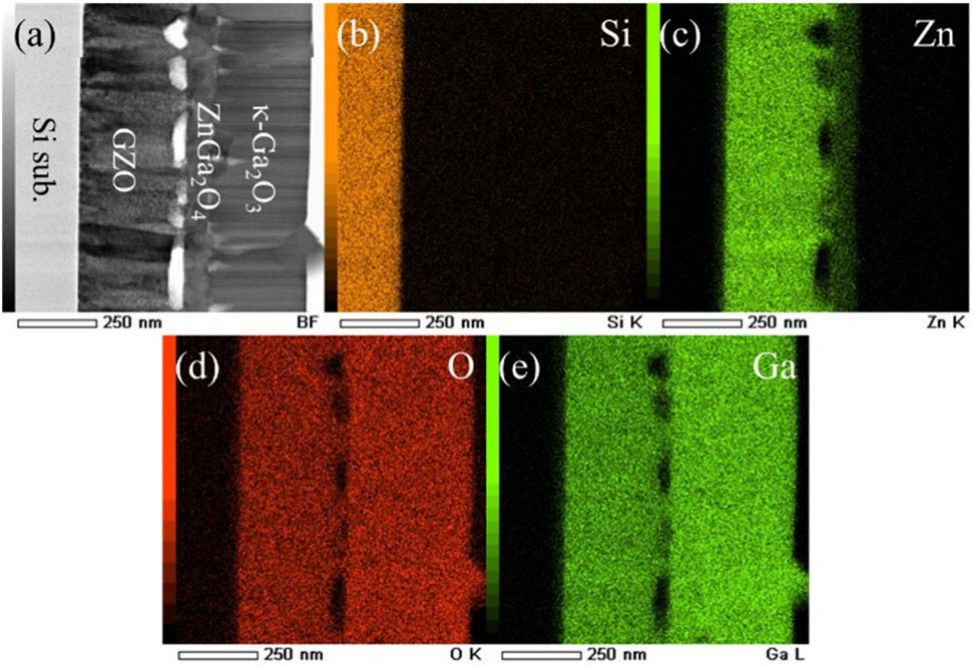
(a) STEM image of κ-Ga_2_O_3_/GZO/Si. (b)–(e) Elemental mapping showing the dispersion of Si, Zn, O, Ga.

Next, the elemental composition of the sample was investigated using STEM/EDX. Fig. 3(a)–(e) shows a scanning transmission electron microscopy (STEM) image of the κ-Ga_2_O_3_/GZO/Si structure and the corresponding elemental maps of Si, Zn, O, and Ga obtained by EDX. As shown in Fig. 3(c), a region with a clearly lower Zn concentration compared with the GZO layer is observed, which corresponds to the ZnGa_2_O_4_ transition layer.

**Fig. 4:**
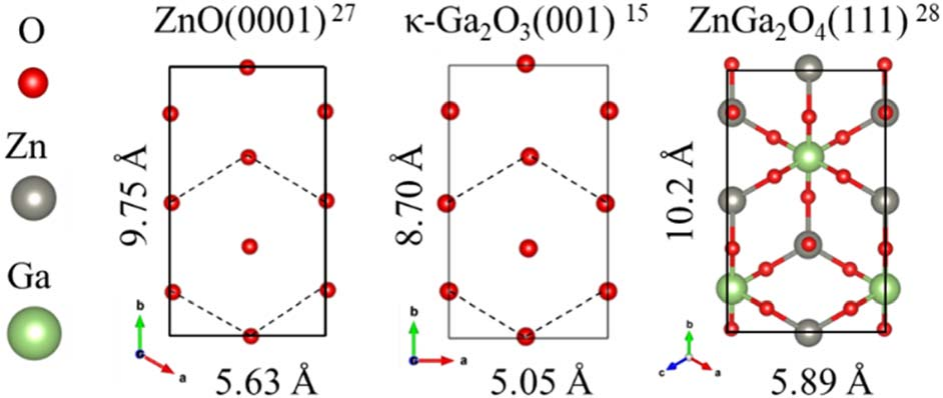
The oxygen atomic arrangements of κ-Ga_2_O_3_(001)^15^ and ZnO(0001),^27^ together with the atomic configuration of ZnGa_2_O_4_(111).^28^

The formation of the ZnGa_2_O_4_ intermediate layer is likely attributed to the reaction between the GZO surface and HCl-containing precursor solution during the mist CVD process. The HCl likely etches the GZO surface, creating a Zn–Ga mixed composition that crystallizes as the spinel-structured ZnGa_2_O_4_ with (111) planes parallel to the substrate. Fig. 4 shows the oxygen atomic arrangements of κ-Ga_2_O_3_(001) and ZnO(0001), together with the atomic configuration of ZnGa_2_O_4_(111). The ZnGa_2_O_4_(111) plane, with its hexagonal atomic arrangement, is likely to maintain compatibility with the hexagonally arranged oxygen atoms on the underlying GZO(001) and overlying κ-Ga_2_O_3_(001), potentially allowing the layer sequence to form without significant structural disruption. The complete orientation relationship is κ-Ga_2_O_3_(001)//ZnGa_2_O_4_(111)//GZO(001)//Si(100), where the double slashes indicate parallel planes but not necessarily epitaxial relationships owing to the polycrystalline nature of the layers.

**Fig. 5:**
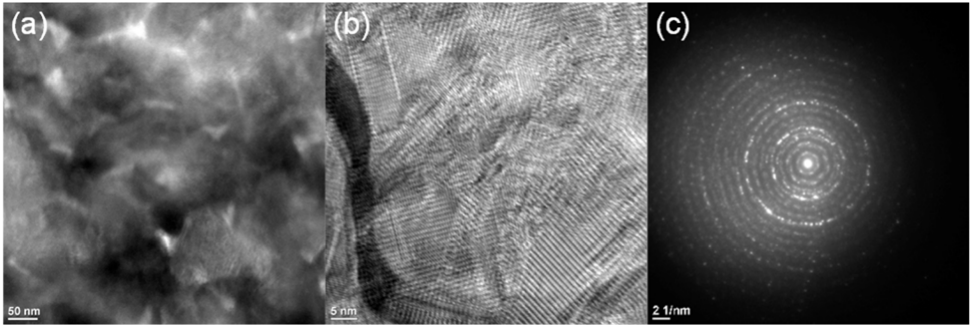
(a) Plan-view TEM image of κ-Ga_2_O_3_ film. (b) High-resolution plan-view TEM image of κ-Ga_2_O_3_. (c) SAED pattern obtained from the plan-view TEM image.

To directly investigate the in-plane orientation distribution, plan-view TEM observations were performed. Fig. 5(a) shows a plan-view TEM image, revealing a polycrystalline microstructure with multiple grains exhibiting different contrasts. The variations in contrast across the image indicate that different grains have different crystallographic orientations, with each grain diffracting differently under the same imaging conditions.

Fig. 5(b) shows a high-resolution plan-view image, where clear lattice fringes are visible from multiple grains. Importantly, adjacent grains display lattice fringes with distinctly different orientations, and the angular relationships between grains do not follow the characteristic 120 rotational pattern observed in epitaxial κ-Ga_2_O_3_ films. This direct observation at the atomic scale confirms that the film consists of randomly oriented polycrystalline grains rather than an ordered rotational domain structure.

Fig. 5(c) shows the SAED pattern obtained from the plan-view TEM images (Fig. 5(a)). In contrast to the single-crystal-like pattern observed in cross-sectional SAED (Fig. 2(e)), the plan-view SAED pattern exhibits circular diffraction rings rather than discrete spots. This circular patten is the definitive signature of polycrystalline materials with randomly oriented grains and provides direct evidence that the κ-Ga_2_O_3_ films comprises grains with random in-plane orientations, despite the strong [001] out-of-plane texture confirmed by XRD and cross-sectional TEM. The combination of high-resolution imaging and SAED unambiguously demonstrates the polycrystalline nature of the films.

This polycrystalline structure with random in-plane orientations contrasts sharply with epitaxial κ-Ga_2_O_3_ films grown on single-crystal substrates, such as sapphire. Epitaxial κ-Ga_2_O_3_ typically exhibit well-defined in-plane orientations with characteristics 120 rotational domains resulting from the pseudo-hexagonal oxygen arrangement of κ-Ga_2_O_3_(001). The epitaxial films shows 12 discrete peaks in XRD φ-scan {122}, corresponding to three sets of four-fold symmetric domains rotated by 120° relative to each other. In exceptional cases, single-domain epitaxial growth with only four φ-scan peaks from {122} has been achieved on ε-GaFeO_3_ substrates, exhibiting the same crystal structure of κ-Ga_2_O_3_ with small lattice mismatches. By contrast, the κ-Ga_2_O_3_ film in this study exhibits neither discrete φ-scan peaks nor discrete SAED spots or ordered lattice orientations in plan-view TEM; however, instead it shows a continuous distribution of in-plane orientations characteristics of polycrystalline materials.

This highly-textured polycrystalline structure originates from the growth on polycrystalline GZO (001) buffer layers. As observed in Fig. 2(b), the GZO layer nucleates with random in-plane orientations on the amorphous SiO_2_ at the Si interface. Each GZO grain maintains *c*-axis orientation perpendicular to the substrate but has a different azimuthal orientation. The κ-Ga_2_O_3_ film likely grows epitaxially on individual GZO grains, with each κ-Ga_2_O_3_ grain through the ZnGa_2_O_4_ intermediate layer, as evidenced by the columnar structure observed in cross-sectional TEM. However, given that the GZO grains have random azimuthal orientations, the κ-Ga_2_O_3_ grains that form on different GZO grains also exhibit random in-plane orientations, resulting in the observed polycrystalline structure with strong [001] out-of-plane texture but random in-plane orientations. This structure differs fundamentally from epitaxial growth on single-crystal substrates, where the substrate defines a unique in-plane orientation for all grains, enabling the formation of ordered rotational domain structures across the entire film.

Since ZnGa_2_O_4_ does not exhibit piezoelectric properties, its formation is expected to degrade the overall piezoelectric response of the film. However, this effect may be mitigated by reducing the thickness of the GZO buffer layer, thereby suppressing Zn diffusion into the Ga_2_O_3_ layer.

## Conclusions

In this study, we successfully demonstrated the growth of polycrystalline κ-Ga_2_O_3_ thin films deposited by mist CVD on Si(100) substrates using GZO buffer layers. The *c*-axis-oriented GZO buffer layer promotes the formation of κ-Ga_2_O_3_ due to its hexagonal oxygen atomic arrangement. Furthermore, the presence of amorphous SiO_2_ on the Si substrate prevents the establishment of an epitaxial relationship in the κ-Ga_2_O_3_/GZO/Si structure, resulting in the polycrystalline growth of κ-Ga_2_O_3_. Unlike previous studies employing epitaxial buffer layers such as AlN or Mo, the use of a polycrystalline GZO buffer layer provides a simpler growth process and inherently isotropic film properties, making it a more practical approach for integrating κ-Ga_2_O_3_-based piezoelectric devices with Si technology. Structural analysis revealed that the κ-Ga_2_O_3_ films exhibit random crystallographic orientations, which inherently provide isotropic properties that are advantageous for piezoelectric device applications. The formation of a ZnGa_2_O_4_ intermediate layer at the κ-Ga_2_O_3_/GZO interface was identified as a key factor in stabilizing the κ-phase. Unlike conventional epitaxial κ-Ga_2_O_3_ films with ordered rotational domains, the polycrystalline nature of our films offers more complete isotropy and potentially simplifies the growth process on technologically important Si substrates. These results open new possibilities for integrating κ-Ga_2_O_3_-based piezoelectric devices with silicon technology. Further optimization of the growth conditions and buffer layer engineering can potentially enable high-performance piezoelectric applications of polycrystalline κ-Ga_2_O_3_ thin films.

## Author contributions

Yoshiaki Hirai: methodology, formal analysis, investigation, data curation, visualization, writing – original draft. Htet Su Wai: investigation. Toshiyuki Kawaharamura: resources, investigation. Noriaki Ikenaga: formal analysis, investigation, resources. Osamu Ueda: formal analysis, investigation. Hiroyuki Nishinaka: conceptualization, project administration, funding acquisition, supervision, resources, writing – review & draft.

## Conflicts of interest

There are no conflicts to declare.

## Data Availability

Data will be made available upon request.
